# Neural basis of self-esteem: social cognitive and emotional regulation insights

**DOI:** 10.3389/fnins.2025.1588567

**Published:** 2025-05-20

**Authors:** Morio Aki, Mami Shibata, Yoshihisa Fujita, Michael Spantios, Kei Kobayashi, Tsukasa Ueno, Takashi Miyagi, Sayaka Yoshimura, Naoya Oishi, Toshiya Murai, Hironobu Fujiwara

**Affiliations:** ^1^Department of Psychiatry, Graduate School of Medicine, Kyoto University, Kyoto, Japan; ^2^Integrated Clinical Education Center, Kyoto University Hospital, Kyoto, Japan; ^3^Advanced Occupational Therapy, Faculty of Human Health Science, Graduate School of Medicine, Kyoto University, Kyoto, Japan; ^4^Organization for the Promotion of Neurodevelopmental Disorder Research, Kyoto, Japan; ^5^Department of Developmental Disorders, National Institute of Mental Health, National Center of Neurology and Psychiatry, Kodaira, Japan; ^6^Human Brain Research Center, Graduate School of Medicine, Kyoto University, Kyoto, Japan; ^7^Artificial Intelligence Ethics and Society Team, RIKEN Center for Advanced Intelligence Project, Tokyo, Japan; ^8^The General Research Division, Osaka University Research Center on Ethical, Legal, and Social Issues, Kyoto, Japan

**Keywords:** self-esteem, resting state functional magnetic resonance imaging, cerebellum, dorsolateral prefrontal cortices, ventrolateral prefrontal cortices

## Abstract

**Introduction:**

Self-esteem (SE) can significantly affect individual well-being and has been linked to various psychiatric conditions. SE involves cognitive and emotional regulation within a social context. Prior research focusing on young adults has indicated neural correlations in prefrontal cortex areas but presented inconsistent findings. Our study expanded this to a broader age range and covariates, and examined the influence of subthreshold depression, emphasizing the functional role of the dorsolateral (dlPFC), ventrolateral prefrontal cortices (vlPFC) and cerebellum in social cognition and emotional regulation of social exclusion.

**Methods:**

We conducted resting-state functional magnetic resonance imaging analyses on 114 participants to investigate the neural correlates of self-esteem.

**Results:**

We found that high SE correlated with robust functional connectivity between the left dlPFC and posterior cerebellum. Associations between the left dlPFC and right lingual gyrus, the right vlPFC and insula were FDR-survived, along with diminished connectivity between the left vlPFC, angular gyri, and thalamus.

**Discussion:**

These results not only support our hypothesis regarding the dual role of SE—which includes its social cognitive role in avoiding social exclusion and its emotional resilience in enduring such exclusion—but also suggest a potential link with rumination.

## 1 Introduction

Global self-esteem (SE), subjective appraisal of self-worth or value (Tafarodi and Swann, 1995) profoundly influences our patterns of social interactions, decision-making processes and social capabilities and mental well-being (Baumeister et al., 2003; Kuster et al., 2013). Recent meta-analysis strengthen the significance of high SE for adaptive social behaviors and mental health (Orth and Robins, 2022). In contrast, insufficient SE is implicated in a multitude of psychiatric conditions or symptoms, such as anxiety (Rosenberg and Owens, 2009; Sowislo and Orth, 2013; Nguyen et al., 2019), eating disorders (Heatherton and Baumeister, 1991; Pelc et al., 2023), self-stigma, suicide (Corrigan et al., 2009; Oexle et al., 2017), and depression (Cheng and Furnham, 2003; Orth and Robins, 2013; Huang, 2021). From a neuroscientific perspective, these psychological phenomena suggest some underlying neural frameworks that SE may modulate, thus warranting the importance of exploration of associated neural mechanisms.

The complex etiology of SE has led to the development of various hypotheses. One influential perspective is the social-cognitive aspect, which highlights how we evaluate ourselves within social contexts. Leary et al. proposed the sociometer theory (Leary et al., 1995a,b), in which SE plays as a psychological “sociometer” or gauge that monitors the risk of social exclusion. A decrease in SE can indicate a potential disruption in social relationships, promoting actions in individuals to avoid social pain or rejection. Conversely, people with high SE are resilient against negative self-related emotions arising from social rejection and reputational concerns. Specifically, individuals with high SE can effectively maintain self-worth and a positive self-image despite experiencing social rejection or failure (Brown and Marshall, 2001).

These contrasting characteristics of SE raised the following important question: “How can brain reconcile SE’s role as a guardian of social inclusion with its ability to mitigate the emotional impact of social setbacks?” From a neurobiological standpoint, social exclusion and inclusion signals may involve neural circuits in prefrontal area. Especially dorsolateral prefrontal cortex (dlPFC) and ventrolateral prefrontal cortex (vlPFC) could be key neural substrates in emotion regulation and responses to social exclusions, because studies have highlighted the involvement of the dlPFC with distraction strategies and the vlPFC in reappraisal strategies during social pain regulation and modulating emotional responses to social exclusion (He et al., 2018; Zhao et al., 2021). Cerebellar regions are also candidates of SE neural basis associated with social cognition (crus 1) and emotional regulation (crus 2) (Sokolov, 2018; Van Overwalle et al., 2020a), and cortico-cerebellar circuit is reported to be altered in people with social anxiety disorder(Zhang et al., 2022), which also validate our focus on the cerebellum. Although regions such as the amygdala and cingulate cortex also contribute to emotional processing, we focused on the PFC regions for their direct involvement in higher-order self-evaluation and regulatory mechanisms central to SE. So as the neural basis of sociometer theory and social setback buffer of SE, we hypothesized dlPFC and cerebellum’s activity mediates social and emotional monitoring.

Neuroimaging research was conducted to clarify the relationship between SE and brain structure and function. Previous voxel-based morphometry (VBM) studies reported a positive correlation between SE and regional volume in areas linked to adaptive stress responses (McEwen and Gianaros, 2010; Agroskin et al., 2014). According to these studies, individuals with lower SE showed decreased gray matter volume in these areas, possibly suggesting difficulties in emotional self-regulation during stress.

Several task-based functional magnetic resonance imaging (fMRI) studies identified increased activity in the medial prefrontal cortex (mPFC), which was correlated with higher SE during self-related social feedback (van der Meer et al., 2010; Chavez and Heatherton, 2015; Yang et al., 2016). Further, two studies reported correlations between SE and activity in the prefrontal cortices of young participants using fractional amplitude of low-frequency fluctuations and resting-state fMRI (rsfMRI) (Pan et al., 2016; Chen et al., 2021). However, the regions of interest and results were inconsistent across studies; one found correlations in the ventromedial prefrontal cortex whereas the other in the right dorsolateral prefrontal cortex (dlPFC).

The inconsistent findings from rsfMRI studies in the PFC and the unexplored influence of age and subthreshold depression on SE require closer attention. Therefore, the present study aimed to bridge these knowledge gaps and provide a clearer understanding of the neural substrates underlying SE.

To explore the neural basis of SE, this study incorporated two important perspectives. First, SE fluctuates and develops across age due to cumulative life events (Erol and Orth, 2011). Thus, a broader age range should be included and covariated to understand SE-related brain connectivity without specific age-range effect (like connectivity trait in adolescent SE). Second, subthreshold depression, which is characterized by mild depressive symptoms that do not meet full diagnostic criteria (Pincus et al., 1999), is frequently associated with lower SE and altered neural functioning. Global SE, measured with the Rosenberg Self-Esteem Scale (RSES), is generally considered a relatively stable trait in healthy individuals (Donnellan et al., 2012). However, previous research reported a correlation between depressive symptoms in healthy participants and SE; for example, in individuals with a negative self-image (interpreted as individuals with lower SE) exhibited depressive symptoms but were not diagnosed with depression (Savilahti et al., 2018). Similarly, SE was found to correlate with depression scales even in healthy participants (Nguyen et al., 2019; Jafari et al., 2021). In a VBM study, similar changes in depression scales were found in individuals with subthreshold depression (Li et al., 2017), while functional changes in the prefrontal regions, such as the right orbitofrontal cortex (OFC) and left dlPFC (Ma Q. et al., 2013; Ma Z. et al., 2013; Wu et al., 2025), along with changes in large-scale networks (Hwang et al., 2015), were also reported in older individuals with subthreshold depression. Overall, these findings highlight the need to exclude the effects of subthreshold depression in a study on the neural basis of SE. However, to the best of our knowledge, SE fMRI studies considering subthreshold depression have not yet been conducted.

Given these theoretical and empirical backgrounds, the present study aimed to elucidate how individual differences in SE are associated with resting-state functional connectivity (rsFC) within networks including prefrontal areas, controlling for age, sex, and subthreshold depressive symptoms. We hypothesized that higher SE would correlate with increased functional connectivity between prefrontal regions (particularly dlPFC and vlPFC) and cerebellar regions involved in social cognition and emotional regulation, reflecting more efficient neural integration supporting positive self-evaluation and emotional resilience.

## 2 Materials and methods

### 2.1 Participants

Overall, 128 healthy adults(mean age = 29.07, 82 males), recruited through advertisements and personal contact, underwent mental health screening by experienced psychiatrists using the Structured Clinical Interview for DSM-IV Disorders (SCID-IV-TR). The exclusion criteria were 1. psychiatric disorders or severe medical or neurological illnesses, 2.claustrophobia or any other reason for unavailability of MRI scan, 3. Abnormal brain structures such as arachnoid cysts and cavum vergae, 4. Incomplete psychological scale, and 5. excessive head motion during scans (details in 2.5 image preprocessing section). After exclusions (see 3.1 Demographic information), 114 participants remained. All participants provided written informed consent. This study was approved by the Ethics Committee of the Faculty of Medicine, Kyoto University Graduate School, and was performed in accordance with the tenets of the Declaration of Helsinki.

### 2.2 Psychological questionnaires

The RSES (Rosenberg, 1965), which measures global SE, encompasses 10 items, each with a 4-point Likert scale, yielding a total score between 10 and 40. Higher scores indicate higher SE. The validated Japanese version was used for the study (Mimura and Griffiths, 2007; Uchida and Ueno, 2010). The internal consistency (Cronbach’s alpha) is = 0.81.

The Beck Depression Inventory-II (BDI-II) (Beck et al., 1996) is a 21-item scale that assesses the severity of depressive states. Scores range from 0 to 63, with higher scores denoting more severe depressive tendencies. The Japanese version has been previously validated and exhibits internal consistency (alpha = 0.87) (Kojima et al., 2002).

### 2.3 Statistical analyses

We performed statistical analyses using SPSS Statistics (version 26.0; IBM, Armonk, NY). Bivariate correlations among key variables (gender, age, SE, depression symptoms, and head motion) were calculated. Spearman’s rank correlation was used for correlation analysis. The correlation was considered statistically significant at *p* < 0.05. Seed-to-voxel FC analysis were conducted to search for the FC correlating SE, using General Linear Model (GLM),with BDI-II scores, age, and gender as covariates. We did not use framewise displacement (FD) as covariates because in the ART-scrubbing procedure FD threshold was set on 0.5 mm and identified and regressed out high-motion frames based on FD values (see 2.5 preprocessing), and subjects with large movement is excluded from the analysis. Therefore, additional inclusion of mean FD as a covariate in FC analyses was considered redundant and not applied. Statistical significance was set as following: an initial voxel-level threshold was set at *p* < 0.001(uncorrected), followed by a cluster-level threshold corrected using false discovery rate (FDR) at p < 0.05. Additionally, a Bonferroni corrected threshold was set at *p* < 0.0041, for multiple comparisons for the 12 ROIs, so it means that results surviving Bonferroni correction (*p* < 0.0041) are considered statistically robust, while result significant only after FDR are exploratory.

### 2.4 Neuroimaging acquisition

MRI was performed using a 3-T MRI scanner (Tim-Trio; Siemens, Erlangen, Germany) with a 40-mT/m gradient and a receiver-only 32-channel phased-array head coil. To acquire a 360-s (6-min) rsfMRI scan, a single-shot gradient-echo planar imaging (EPI) pulse sequence was used. The participants were instructed to look at a fixation cross in the center of the screen and avoid thinking about anything during the resting-state data acquisition. Head movement was minimized by placing rubber pads within the head coil. For B0 field-mapping distortion correction, a dual-gradient echo dataset was also acquired. Magnetization-prepared rapid gradient-echo (MPRAGE) sequences were used to acquire T1-weighted three-dimensional structural images. Data were excluded from the analysis if structural abnormalities, such as arachnoid cysts or cavum vergae, were detected.

The parameters used were as follows: MPRAGE: echo time (TE), 3.4 ms; repetition time (TR), 2,000 ms; inversion time, 990 ms; field of view (FOV), 225 × 240 mm; matrix size, 240 × 256; resolution, 0.9375 × 0.9375 × 1.0 mm^3^; and 208 total axial sections without intersection gaps; rsfMRI: TE, 30 ms; TR, 2,500 ms; flip angle, 80°; FOV, 212 × 212 mm; matrix size, 64 × 64; in-plane spatial resolution, 3.3125 × 3.3125 mm^2^; 40 total axial slices; slice thickness, 3.2 mm with 0.8-mm gaps in ascending order.

### 2.5 Image preprocessing

To rectify EPI distortions in the rsfMRI dataset, FMRIB’s Utility for Geometrically Unwarping EPIs was employed, a component of the FSL software suite (FMRIB’s software library version 5.0.9). This process included alignment adjustments using FMRIB’s Linear Image Registration Tool (FLIRT). We removed artifactual components and movement-related fluctuations using the FMRIB ICA-based x-noiser (Griffanti et al., 2014). FD was calculated to assess head motion during rsfMRI (Power et al., 2012), using the ART-based scrubbing procedure implemented in CONN toolbox. The following two exclusion criteria were applied based on past rsfMRI studies: (exclusion criteria 5–1) when the count of scans showing a head position variance of 0.5 mm from neighboring scans surpassed 25% and (5–2) when the peak head motion exceeded 3.0 mm or a 3.0° angle (Nilsonne et al., 2017; Zhang et al., 2015).

The CONN-fMRI Functional Connectivity toolbox (version 22.a) (Whitfield-Gabrieli and Nieto-Castanon, 2012), and the statistical parametric mapping software package SPM12 (Wellcome Trust Centre for Neuroimaging) were employed for the processing of the preprocessed rsfMRI and structural MRI data. Using a bespoke preprocessing pipeline of the CONN toolbox, all functional images were entailed with spatial normalization into the standard Montreal Neurological Institute (MNI) space, resampled to 2 mm isotropic voxels, underwent outlier detection through ART-based scrubbing, and were smoothed utilizing a Gaussian kernel having a full-width-at-half maximum of 8 mm. A standard preprocessing pipeline for volume-based analysis was used for all preprocessing steps. The same pipeline was used to segment and normalize the structural data into gray matter, white matter (WM), and cerebrospinal fluid (CSF). The principal components of the WM and CSF signals, along with translational and rotational motion parameters (including six additional parameters signifying their first-order temporal derivatives) obtained from realignment using FLIRT, were eliminated using the CONN covariate regression analysis. Standard denoising pipeline was used to minimize the impact of confounding covariates, including the CompCor strategy (Behzadi et al., 2007), which extracted fluctuations in the rsfMRI signals originating from the WM and CSF, along with their derivatives, and noise stemming from realignment parameters. Additionally, bandpass filtering was executed. The frequency window was 0.008–0.09 Hz. This processing step resulted in an increase in retest reliability.

### 2.6 FC analysis

**Figure 1:**
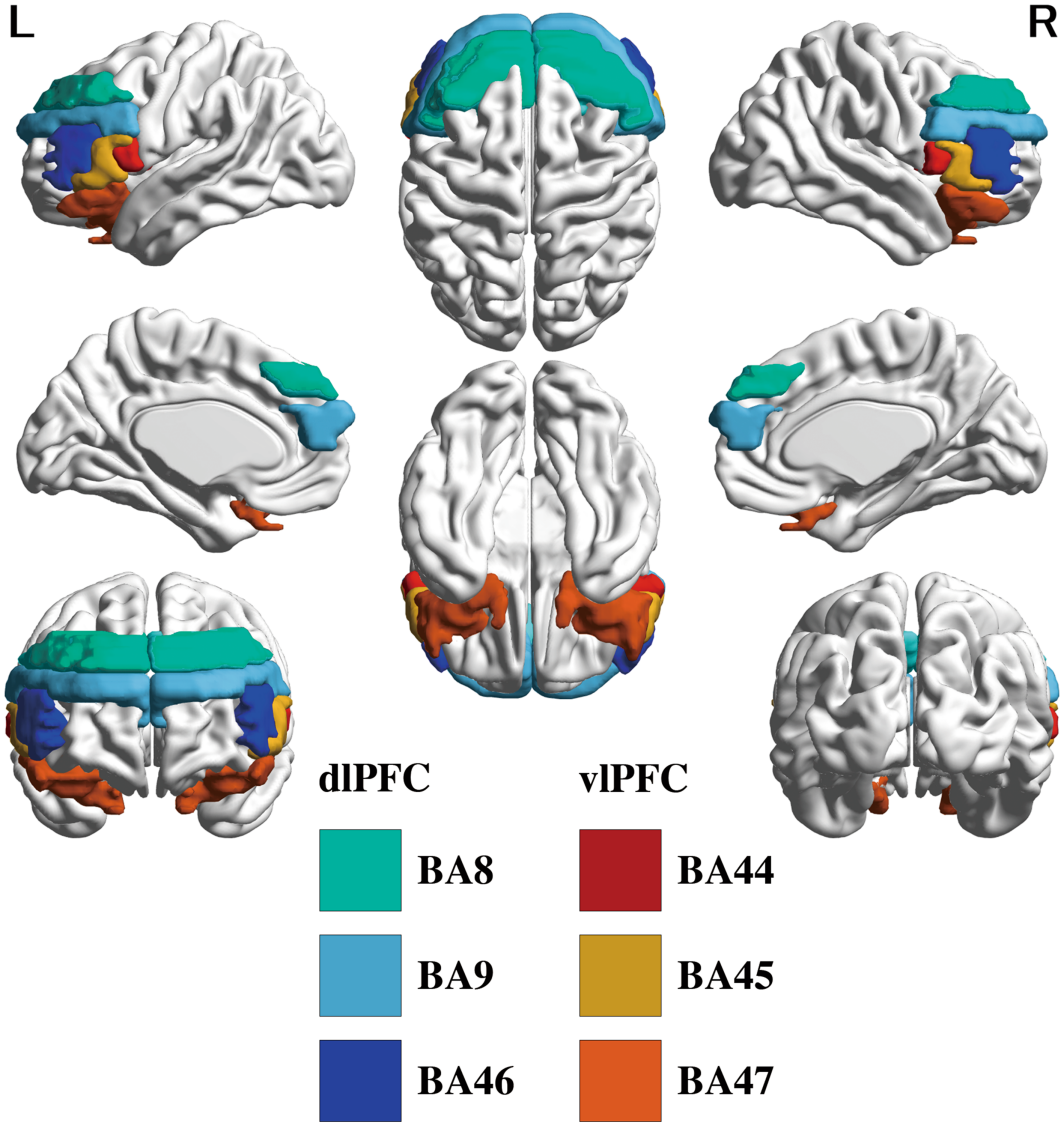
Regions of interest that are targeted. The DLPFC (BA 8, 9, 46) and VLPFC (BA 44, 45, 47) are colored in the glass brain. DLPFC, dorsolateral prefrontal cortex; VLPFC, ventrolateral prefrontal cortex; BA, Brodmann area.

Seed-to-voxel rsFC analyses were conducted to examine FCs correlating with SE. The regions of interest (ROIs) were the dlPFC and vlPFC. We used the Brodmann area (BA) atlas to define these areas anatomically. Employing the BA atlas in CONN (path: util/otherrois/BA.img), which was transformed into the MNI space via a Lancaster transformation from the Talairach atlas, we established seeds on bilateral BA 8 (frontal eye fields), 9 (dorsolateral and medial prefrontal cortex), and 46(anterior middle frontal gyrus and middle frontal area) for the dlPFC (Sallet et al., 2013) and 44 (opercular part of inferior frontal gyrus: IFG), 45 (triangular part of IFG), and 47 (orbital part of IFG) for the vlPFC (Levy and Wagner, 2011), totaling 12 seeds for analysis (Figure 1). Based on the initial FC analysis results, we conducted a post-hoc seed-to-voxel analysis using cerebellar seeds (bilateral crus 1, 2, and lobule 6, which were included in the Bonferroni-survived cluster, and were reported regions to be involved in social or emotional recognition: Van Overwalle et al., 2020b) from the the Automated Anatomical Labeling atlas provided in CONN toolbox. This post-hoc analysis is to specify the functional meaning and region of the results of the main analysis.

## 3 Results

### 3.1 Demographic information

After completing all psychological tests and MRI examinations, 14 participants were excluded from the analysis. No participants were excluded by the psychiatric diseases (criteria 1) and claustrophobia (criteria 2), but six participants had minor organic brain abnormalities (arachnoid cysts and cavum vergae: criteria 3), four of them did not fully answer the psychological tests(criteria 4), and MRI images of four participants had a maximum motion of more than 3 mm or 3°(criteria 5). Consequently, 114 participants’ data were included in the analysis.

Their demographic data are summarized in Table 1. The RSES seems low in average, but the score is not so different from the past research (e.g., Schmitt and Allik, 2005). Regarding the BDI-II scores, 16 participants had a score of 14 or more, which is the threshold for mild depression, as defined by Beck and the Japanese version of the BDI-II (Hiroe et al., 2005), indicating that these participants were considered healthy on the SCID-IV-TR but had values above the threshold for mild depression on the BDI-II. Their average of FD was 0.12 mm(SD = 0.05), which is in a normal range(Power et al., 2012).

**Table 1:** Demographic and clinical characteristics.

Baseline characteristics	Mean ± SD
Mean age (in years)	29.66 ± 12.56
Sex (female/male)	42/72
Rosenberg’s self-esteem scale (mean)	26.9 ± 4.7
Beck depression inventory - II (mean)	7.0 ± 6.7
Framewise displacement (mean)	0.12 ± 0.05

### 3.2 Analysis of psychological data

We conducted Spearman’s correlations among age, gender, RSES scores, BDI-II scores, and mean FD. Significant correlations were observed between age and FD (*ρ* = 0.24, *p* = 0.01), indicating that older participants had more head motion. Additionally, BDI-II score was negatively correlated with the RSES score (*ρ* = −0.45, *p* < 0.001), suggesting that the participants with higher SE scores have lower depressive symptoms. There were no significant correlations between gender and other variables, nor between FD and psychological measures(all *p* > 0.05). The whole result is shown in Supplementary Table 1.

### 3.3 Main result of functional connectivity related to SE

We performed a correlation analysis of the functional connectivity (FC) strength to explore the neural correlates of SE. We found four seeds (BA 8 left, BA 9 left, BA 44 left, and BA 45 right) that had statistically significant FC values correlating with RSES scores in seed-to-voxel analyses; however, significant correlations were observed only in the left BA 9 after correcting for multiple comparisons (Table 2).

**Table 2:** Summary of seed-to-voxel analyses.

Seed	Anatomical location (BA) of cluster	L/R	Cluster MNI space, x,y,z	Cluster size	Size ρ (FDR-corrected)
BA8 left	Lingual gyrus (BA18,19) and cerebellum lobule 6	Right	+12, −62, −24	471	0.00605
BA9 left	Cerebellum crus I, II and lobule 6	Left	−22, −70, −30	634	0.000834*
	Cerebellum crus I, II, and lobule 6	Right	+18, −70, −26	322	0.0209
BA44 left	Thalamus and brain stem	Left	+12, −32, −04	365	0.0344
	Angular gyrus (BA40)	Left	−32, −74, +34	235	0.05
	Angular gyrus (BA40)	Right	+32, −54. +34	228	0.05
	Frontal pole (BA10)	Right	+26, +54, −14	305	0.0358
BA45 right	Frontal orbital cortex (BA47) and insular cortex (BA13)	Right	+40, +20. −10	396	0.0165

* Survived Bonferroni multiple comparison (*ρ* < 0.0041): the other clusters are only FDR-survived and exploratory results. BA, Brodmann area; FDR, false discovery rate, MNI, Montreal Neurological Institution.

**Figure 2:**
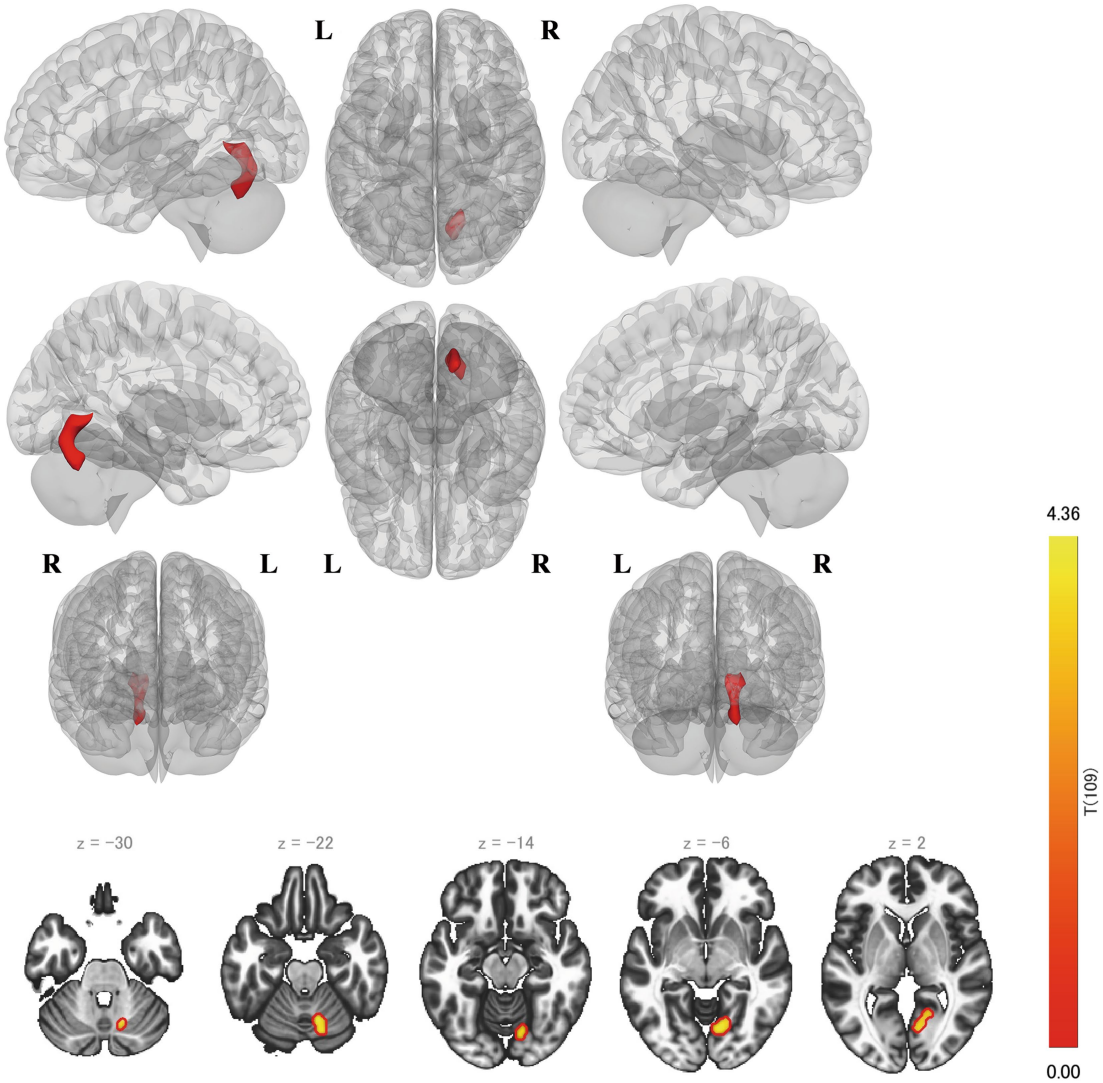
Results of the seed-to-voxel analysis on the left BA 8. The upper image is a glass brain view, and the lower image is a slice view. In the slice view, the positively correlated clusters are circled red, and the negative clusters are blue. The color bar shows the t-value, and the degree of freedom is 109 (same as in Figures 1–5). BA, Brodmann area.

The left BA 8 seed yielded a significant positive correlation with the RSES score in the rsFC with the right lingual gyrus (BA 18, 19) and right cerebellar lobule 6 (*p* = 0.0061) (Figure 2).

**Figure 3:**
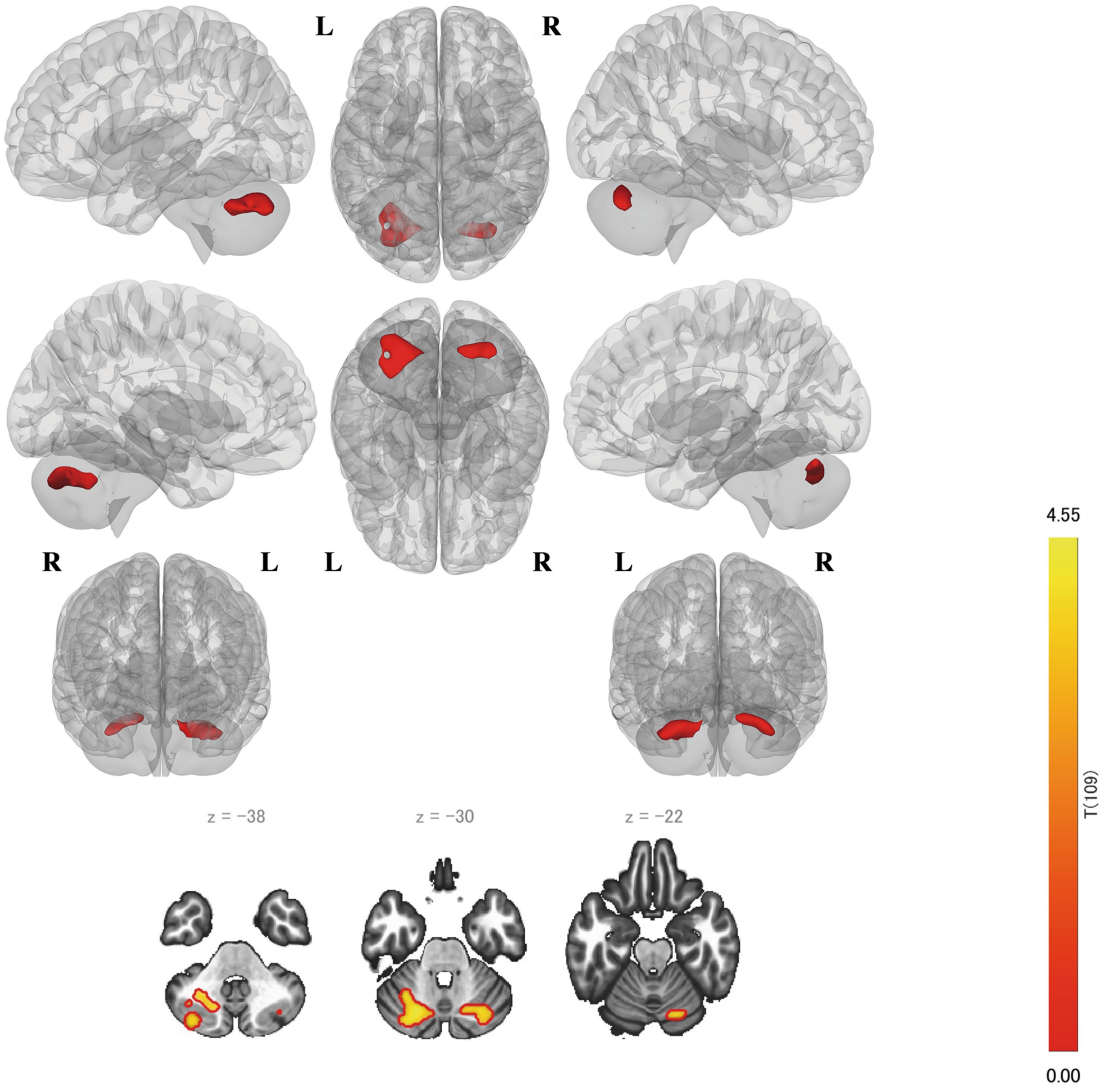
Results of the seed-to-voxel analysis on the left BA 9. The left cluster remains even after Bonferroni multiple correction is considered. BA, Brodmann area.

The FC strength between the left BA 9 and bilateral cerebellum was significantly positively correlated with the RSES scores (left: *p* = 0.0008, right: *p* = 0.0209) (Figure 3). Both clusters primarily covered crus 1 and 2 and lobule 6 of each cerebellar hemisphere. Cluster 1 on the left cerebellum was the largest and was the only cluster that was significantly correlated after multiple corrections were applied. Notably, the right side of dlPFC showed no significant FC (Table 2).

**Figure 4:**
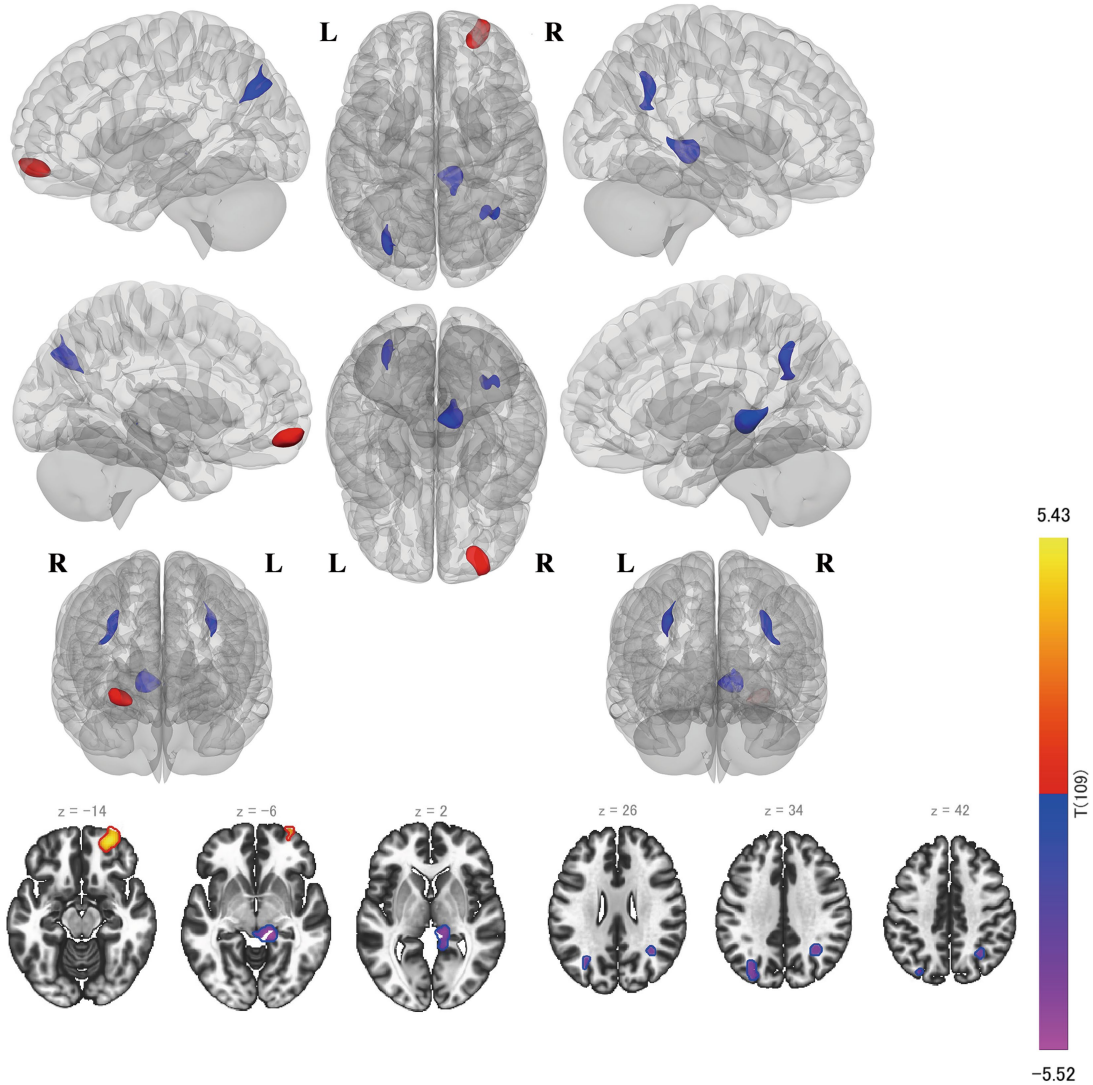
Results of the seed-to-voxel analysis on the left BA 44. The blue clusters are negatively correlated with SE, and the red clusters are positively correlated with SE. BA, Brodmann area; SE, self-esteem.

**Figure 5:**
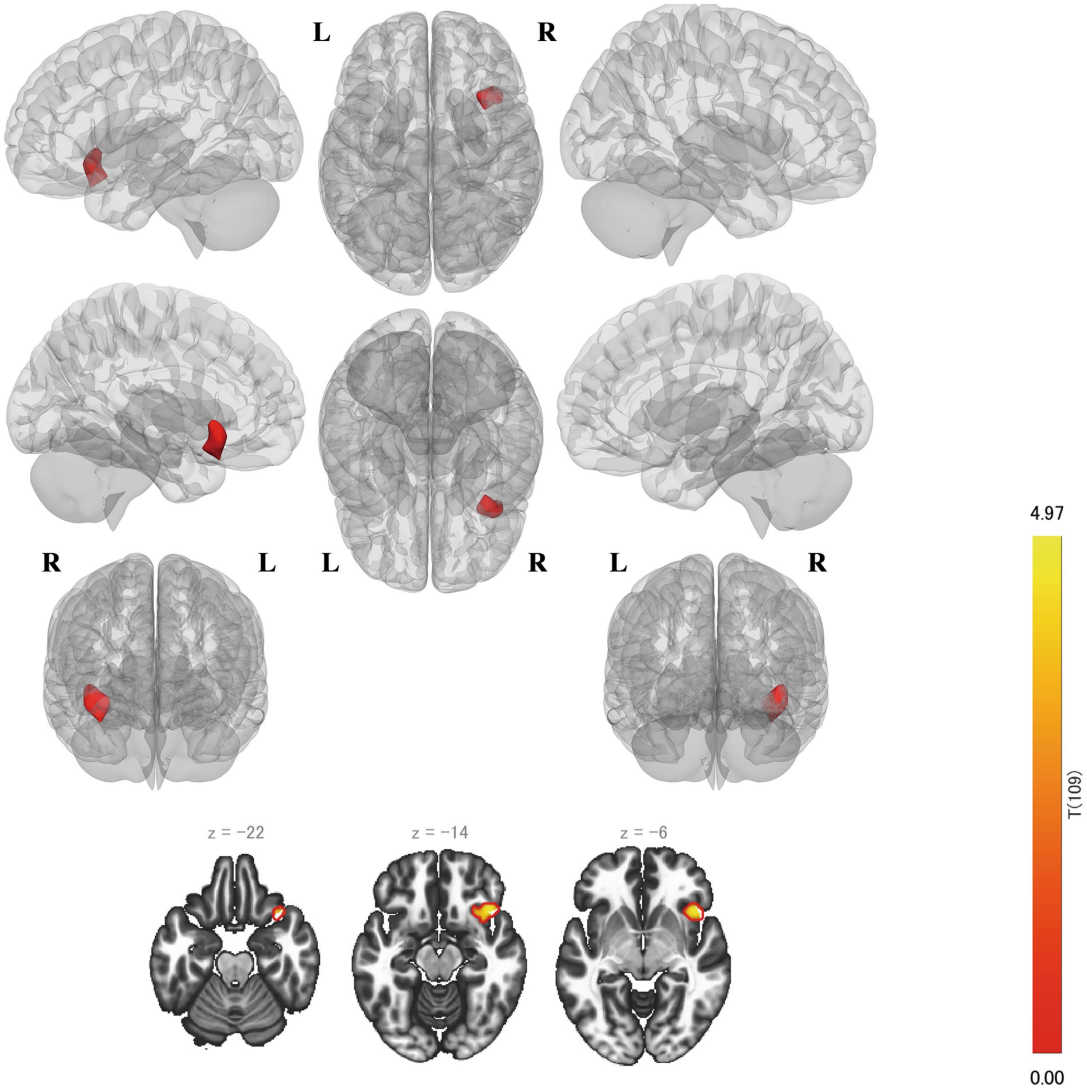
Results of the seed-to-voxel analysis on the right BA 45. BA, Brodmann area.

The left BA 44 yielded four clusters (Figure 4). Three of them were located on the thalamus, brainstem, and both sides of the angular gyrus, which were negatively correlated with the RSES score, while the other one was located on the right frontal pole (BA 10) and was positively correlated with the RSES. The right BA 45 seed yielded a positive correlation with the RSES, including the right frontal orbital cortex (BA 47) and right insular cortex (BA 13) (Figure 5).

### 3.4 Post-hoc analysis to understand the part of significant FC, seeding on the cerebellum

We got the result on BA8 and 9 on the main analysis, but that seed is spatially widely spread from medial to lateral (see Figure 1).To understand which side of (medial or lateral) BA 8 and 9 had connectivity with the cerebellum and to consider the functional significance, we performed a post-hoc seed-to-voxel analysis with the cerebellar seeds (according to the result in BA8 and 9, we set the ROI in both side of Crus 1, 2, and lobule 6). Both sides of the cerebellum crus 1 had a significant connection with the lateral prefrontal area. The left cerebellum crus 1 yielded a cluster correlating with the RSES, including the frontal pole cortex (BA 10) and dlPFC (BA 9) (Supplementary Figure S1). The right side of crus 1 showed significant connectivity with both sides of the lateral anterior prefrontal area. The two clusters were both mainly included BA 9 and 10 (Supplementary Figure S2). The detailed results of clusters are shown in Supplementary Table 2.

## 4 Discussion

### 4.1 Summary of main findings

This pioneering rsfMRI study probed the neural underpinnings of SE in 114 healthy adults, meticulously considering and covarying out the potential influence of age, sex, and depressive symptoms. These factors can subtly vary within a healthy population and could confound the interpretation of the FC between brain regions related to social cognition and emotional regulation.

Before discussing the main FC findings, our bivariate analyses revealed a negative correlation between the RSES and BDI-II scores, which underscores the close link between SE and depressive symptoms, even among non-clinical populations. This correlation aligns with previous research demonstrating that individuals with higher BDI-II scores harbor a more potent negative self-image, even without major depression (Vaccaro et al., 2017). Importantly, this result substantiates the validity of our decision to statistically control for depressive symptoms. Additionally, a negative correlation between age and mean FD was observed, consistent with the previous reports (Hausman et al., 2022; Kato et al., 2021), further supporting covarying age in our rsfMRI analyses.

SE is postulated to relate closely to the sociometer theory, implicating brain regions responsible for social cognition and emotional regulation. Our findings support this hypothesis, particularly emphasizing the robust increase in FC between the left dlPFC and the posterior cerebellum. This result survived strict multiple comparison corrections, and suggests a significant neural link potentially involved in the integration of social cognitive inputs and emotional processing with the cerebellum. In addition to this central finding, exploratory results indicated that enhanced FC between the left dlPFC and the lingual gyrus might reflect integrated self-referential emotional and cognitive processing, whereas increased FC within the right vlPFC could relate to emotional regulation. Among these, the robust FC between the dlPFC and posterior cerebellum merits particular attention regarding the neural basis of SE.

### 4.2 FC between dlPFC and cerebellum positively correlated with SE: implications for social cognition and emotional regulation

Enhanced FC was observed primarily between the dlPFC regions (BA8 and 9) and posterior cerebellum (crus 1and 2, lobule 6). Post-hoc analyses further supported increased FC between cerebellar crus 1 and lateral, not medial, part of prefrontal cortexes including dlPFC and anterior prefrontal cortes (aPFC). Here we review the role of each region and interpret this FC correlation with SE.

#### 4.2.1 dlPFC

The dlPFC is critically involved in higher-level cognition functions (O’Reilly, 2006), coordination of responses to environmental stimuli (O’Reilly, 2010), attention control (Lai, 2021), emotional evaluation (Hutcherson et al., 2005), cost-sensitive decision-making (Botvinick and Braver, 2015), social cognitive processing (Weissman et al., 2008), and volition (Nitschke and Mackiewicz, 2005). This region exhibits hemispheric imbalance in depression (Mayberg, 2003; Phillips et al., 2003), with decreased left-side activity associated with depressive symptoms (Dutta et al., 2014) and increased ruminative thought process (Cooney et al., 2010; Wang et al., 2015; Lefaucheur et al., 2020; Baeken et al., 2021).

#### 4.2.2 aPFC

The aPFC (BA 10), or frontal pole, contributes to introspection, self-related processes and social relationships, recording actions, distinguishing real from imaginary events (Tsujimoto et al., 2011), confidence in judgments (Miyamoto et al., 2018), metacognition (Baird et al., 2013), emotional action control (Koch et al., 2018) and executing control beyond automatic behaviors (Volman et al., 2011; Bramson et al., 2020). One study showed that a higher aPFC baseline activity correlated with lower post-traumatic stress disorder symptoms (Kaldewaij et al., 2021), highlighting its role in adaptive emotional and cognitive control.

#### 4.2.3 Cerebellum

Anatomical tracing studies have demonstrated closed-loop circuits between the lateral prefrontal cortex and cerebellar Crus 1 and 2. These circuits are segregated from motor loops, indicating that a substantial portion of the cerebellum is dedicated to cognitive-affective networks rather than sensorimotor control (Habas et al., 2009; Buckner et al., 2011; Habas, 2021). Crus 1 predominantly supports social cognitive processes such as mentalizing and predicting social sequences, whereas Crus 2 is more strongly involved in emotional regulation processes (Guell et al., 2018; Van Overwalle et al., 2020b). These cerebellar regions activate in conjunction with medial prefrontal and temporoparietal areas during theory-of-mind tasks, reflecting their role in internal models of social dynamics and predictive coding of social behaviors (Van Overwalle et al., 2014; Olivito et al., 2023).

Schmahmann (1998) observed psychiatric symptoms in patients with cerebellar damage, proposing the “dysmetria of thought” hypothesis, linking cerebellar impairments in emotional and social cognitive control to psychiatric symptoms such as anxiety, depression, aggression, and passivity (Schmahmann et al., 2007; Hoche et al., 2016). Subsequent research supports cerebellar involvement in emotional processing, predictive coding, and internal modeling of cognitive-affective processes (Alalade et al., 2011; Liu et al., 2012; Guo et al., 2013; Ma Q. et al., 2013; Ma Z. et al., 2013; Baetens et al., 2014; Van Overwalle et al., 2014; Van Overwalle et al., 2015; Depping et al., 2018; Guell et al., 2018; Guell and Schmahmann, 2020).

#### 4.2.4 Interpretation of the positive correlation of FC with SE

Our results showed that higher SE is associated with stronger FC between the dlPFC-aPFC and cerebellar regions. This connectivity likely underpins effective predictive coding and emotional regulation through fronto-cerebellar loops. The dlPFC conveys contextual social information to the cerebellum, which generates prediction regarding cognitive and emotional outcomes. Any mismatch between predicted and actual outcomes results in feedback signals that update and optimize prefrontal processing (Ito, 2008; Sokolov et al., 2017). Weakened connectivity between gradualてdlPFC-aPFC and cerebellar areas could decrease the ability to predict and regulate emotional and social outcomes effectively, thus increasing vulnerability to maladaptive emotional reactions and ruminations (Cooney et al., 2010; Wang et al., 2015).

Conversely, individuals with higher SE may have increased dlPFC-cerebellar interactions that enables efficient monitoring of social feedback, prediction of emotional states, and rapid error correction when social outcomes deviate from expectations. Such adaptive fronto-cerebellar coupling, potentially mediated by interactions with large-scale networks such as the DMN, supports resilience against negative self-evaluations and facilitates stable self-esteem by continuously adjusting social-cognitive strategies and emotional responses (Brown and Mankowski, 1993; Campbell et al., 1991). Thus, this dlPFC-cerebellar network is important not merely for cognitive and affective adjustment but also for sustaining psychological well-being through predictive control in social and emotional dynamics.

### 4.3 Other FC changes which could not survive multiple comparisons (exploratory results)

#### 4.3.1 FC increase between the left BA 8 and right lingual gyrus

We observed a positive correlation between the SE and FC involving the left BA 8 and the right lingual gyrus. The lingual gyrus plays a pivotal role in visual perception, visual agnosia (Hirayama, 2017), facial emotion recognition (Kitada et al., 2010), and self-referential processes (Kircher et al., 2000; Makino and Ikoma, 2022). A reduction in the surface area of the right lingual gyrus has been associated with anxiety, depression (Couvy-Duchesne et al., 2018), and adolescent major depressive disorder (Schmaal et al., 2016), which may influence adult psychiatric outcomes. Based on sociometer theory, cooperation between the lingual gyrus and dlPFC may facilitate integrated processing of self-referential emotional and cognitive information, essential for maintaining positive self-evaluation.

#### 4.3.2 FC increase between the left BA 44 and right frontal pole as well as between the right BA 45, BA 47, and insula

Our analysis identified positive correlations between the left BA 44 and the right frontal pole (right BA 10), as well as between the right BA 45 and a cluster including the right OFC (right BA 47 right) and insular cortex (BA 13).

BA 44, 45, and 47 collectively form the inferior frontal gyrus (IFG), crucial for verbal motor function, working memory (Osaka, 2007), and behavioral regulation (Forstmann et al., 2008; Sundby et al., 2021). The right vlPFC is linked to social exclusion (Riva et al., 2012) and the cognitive reappraisal and suppression of amygdalar activity associated with fear and anger (Lieberman et al., 2007). Previous research detected diminished right IFG activity in depressed individuals experiencing pronounced rumination (Kühn et al., 2012) and increased vlPFC-amygdala FC in individuals with high SE during mortality threat tasks (Yanagisawa et al., 2016), highlighting its importance in emotional and cognitive regulation relevant to SE. One study (Kim G. W. et al., 2022; Kim H. et al., 2022) revealed reduced FC between the bilateral IFG regions in young adults with pronounced suicidality, which is consistent with our findings. The insular cortex is also instrumental in emotional judgment (Zaki et al., 2012). Given the amplified FC among vlPFC, frontal pole, and insula, individuals with high SE may exhibit superior skills in effectively monitoring and regulating emotional states, using the frontal pole as a protective buffer against intense negative emotions.

#### 4.3.3 FC decrease between the BA 44, angular gyri, and thalamus

Seeding on the BA 44 left, a negative correlation with SE was observed in FC involving clusters within bilateral the angular gyri and the thalamus. The left BA 44 (Broca’s area) contributes to the production of language, grammar, fluency, and processing of sentences (Grewe et al., 2005; Ardila, 2012). These are known to work as networks with Wernicke’s area and extended area, including the thalamus (Bohsali et al., 2015; Kim G. W. et al., 2022; Kim H. et al., 2022; Junker et al., 2023). Previous studies have suggested that rumination is negatively associated with SE and is often experienced as inner speech (Moffatt et al., 2020; Bugay-Sökmez et al., 2023), indicating that people with low SE tend to engage in excessive internal verbalization and cognitive rumination during the resting state.

#### 4.3.4 Null findings in right dlPFC seeds

Interestingly, we did not find significant FC correlations with SE involving the right dlPFC(BA8,9,46). The null significant results in the right dlPFC might indicate a left-lateralized neural substrate for SE processing. Prior neuroimaging studies showed greater left-hemispheric prefrontal activation during positive emotional experiences and self-referential cognition (Weissman et al., 2008) Future research should explicitly examine lateralization patterns in neural mechanisms underpinning SE to further validate this hemispheric distinction.

### 4.4 Neural correlates of SE

With multiple comparisons corrections, only FC between the dlPFC and cerebellum remained significant. This robust result suggests the dlPFC’s coordinating role in cognitive process and reciprocal interactions with the cerebellum during SE establishment. Additionally, observed the FC with the lingual gyrus is associated with self-perception and social interaction, while FC among the vlPFC, frontal pole, and insula contributes to emotion regulation. Consequently, our findings suggest the potential for the language network (BA44-angular gyrus-thalamus) to become less active, achieving a state close to true resting conditions without inner speech.

The relationship between SE and FC could serve as a surrogate marker for predicting the risk of mental illnesses onset. Considering that established psychiatric interventions like cognitive-behavioral therapy impact SE (Mann et al., 2004; Roberts, 2006; Niveau et al., 2021), the FC-SE relationship may offer valuable insights for evaluating the effectiveness of these interventions.

### 4.5 Limitations and future prospects

This study has some limitations. First, we only used data from the Japanese population. Previous research indicates that SE varies across countries (Schmitt and Allik, 2005). Despite this, we believe our findings reflect universal aspects of SE. Future research involving international participants for studying SE and brain function is necessary for generalizing present results.

Second, we identified associations between SE and FC in regions involved in social cognition and emotional regulation, but this does not confirm that social cognition or emotional regulation directly mediate or causally influence the neural relationship with SE. Although interpretations were provided regarding these processes, we did not empirically test their direct functional roles. Future studies should explicitly examine the mediating or moderating effects of social cognitive and emotional regulation factors using task-based fMRI paradigms and mediation or moderation analyses (e.g., see Zhang et al., 2023).

Third, this was a cross-sectional study. While SE is relatively stable, it is also known to change gradually over the course of a person’s life. Future studies should observe the correlation between intra-individual changes in SE and changes in FC.

Lastly, we focused on specific seeds to identify SE neural correlates. Although we found some FCs potentially related to large-scale networks like default-mode, these networks were not directly analyzed. Further research should explore the relationship between intra-network and inter-network FC and SE, which might have implications for psychological traits and brain changes.

In conclusion, our study examined the neural correlates of SE, excluding associations with subthreshold depressive symptoms. As hypothesized, FC in social cognition and emotional regulation areas, especially the dlPFC and the posterior cerebellum, was associated with SE. Based on these findings, we propose the possibility of SE as a surrogate marker of mental health management, and the viability of FC as a neurobiological index for evaluating mental health interventions.

## Data Availability

The datasets generated during this study are available from the corresponding author, HF, upon reasonable request.
