# Supplementary material for: Neural basis of self-esteem: social cognitive and emotional regulation insights
Source: Front Neurosci. 2025 May 20;19:1588567. doi: 10.3389/fnins.2025.1588567 (PMC12130037; doi:10.3389/fnins.2025.1588567)
Supplement: Supplementary file 1 [file Data_Sheet_1.docx]

Supplementary Material

**Supplementary Table 1.** The correlation analysis between each demographic data and framewise displacement

| Variable 1 | Variable 2 | Spearman’s ρ | p-value |
| --- | --- | --- | --- |
| Age | Gender | -0.18 | 0.06 |
| Age | RSES | 0.10 | 0.31 |
| Age | BDI | -0.12 | 0.21 |
| Age | FD | 0.24 | 0.01* |
| Gender | RSES | -0.14 | 0.14 |
| Gender | BDI | -0.05 | 0.59 |
| Gender | FD | 0.07 | 0.48 |
| RSES | BDI | -0.45 | 0.00* |
| RSES | FD | 0.09 | 0.37 |
| BDI | FD | -0.07 | 0.45 |

* Significant in p<0.05 level. RSES : Rosenberg’s Self-Esteem Scale, BDI : the Beck Depression Inventory -II, FD : Framewise Displacement

| **Supplementary Table 2.** Summary of post-hoc seed-to-voxel analyses on the cerebellum | | | | | |
| --- | --- | --- | --- | --- | --- |
| Seed | Anatomical location (BA) of cluster | Left/Right | Cluster MNI space, x,y,z | Cluster size | size- P (FDR-corrected) |
|  |  |  |  |  |  |
| Crus1 left | Frontal pole cortex (BA10) and dlPFC (BA9) | Left | -38, +58, +10 | 377 | 0.0277 |
|  |  |  |  |  |  |
| Crus1 right | Frontal pole cortex (BA10) and dlPFC (BA9) | Left | -28, +42, +18 | 765 | 0.000364 |
|  | Frontal pole cortex (BA10) and dlPFC (BA9) | Right | +28. +48, -10 | 518 | 0.002 |
| BA: Brodmann area, dlPFC: dorsolateral prefrontal cortex, FDR: false discovery rate, MNI: Montreal Neurological Institution | | | | | |

**
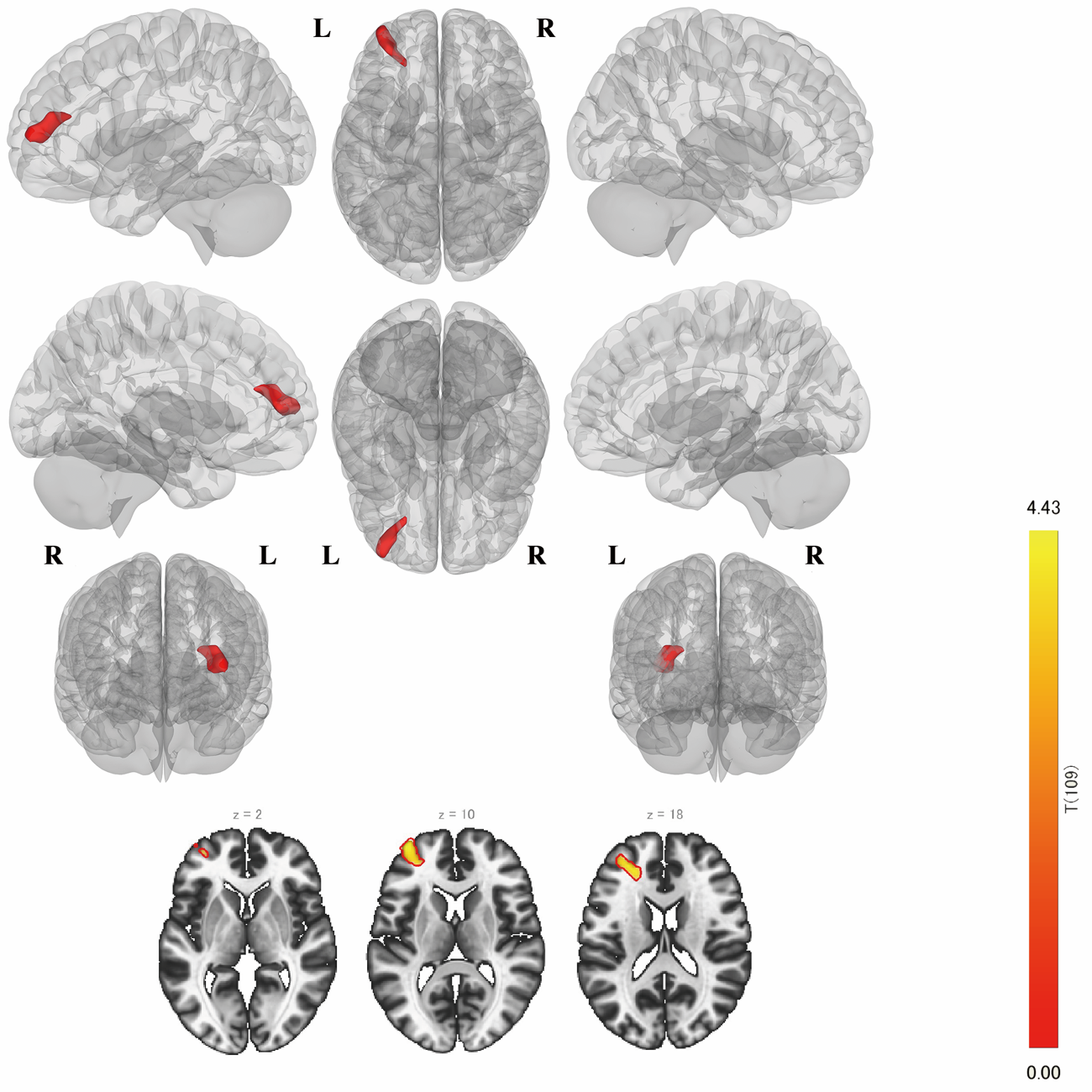
**

**Supplementary Figure 1. Result of the seed-to-voxel analysis on the cerebellum left crus 1.**

The cluster located at left and lateral, not medial , part of prefrontal cortex.


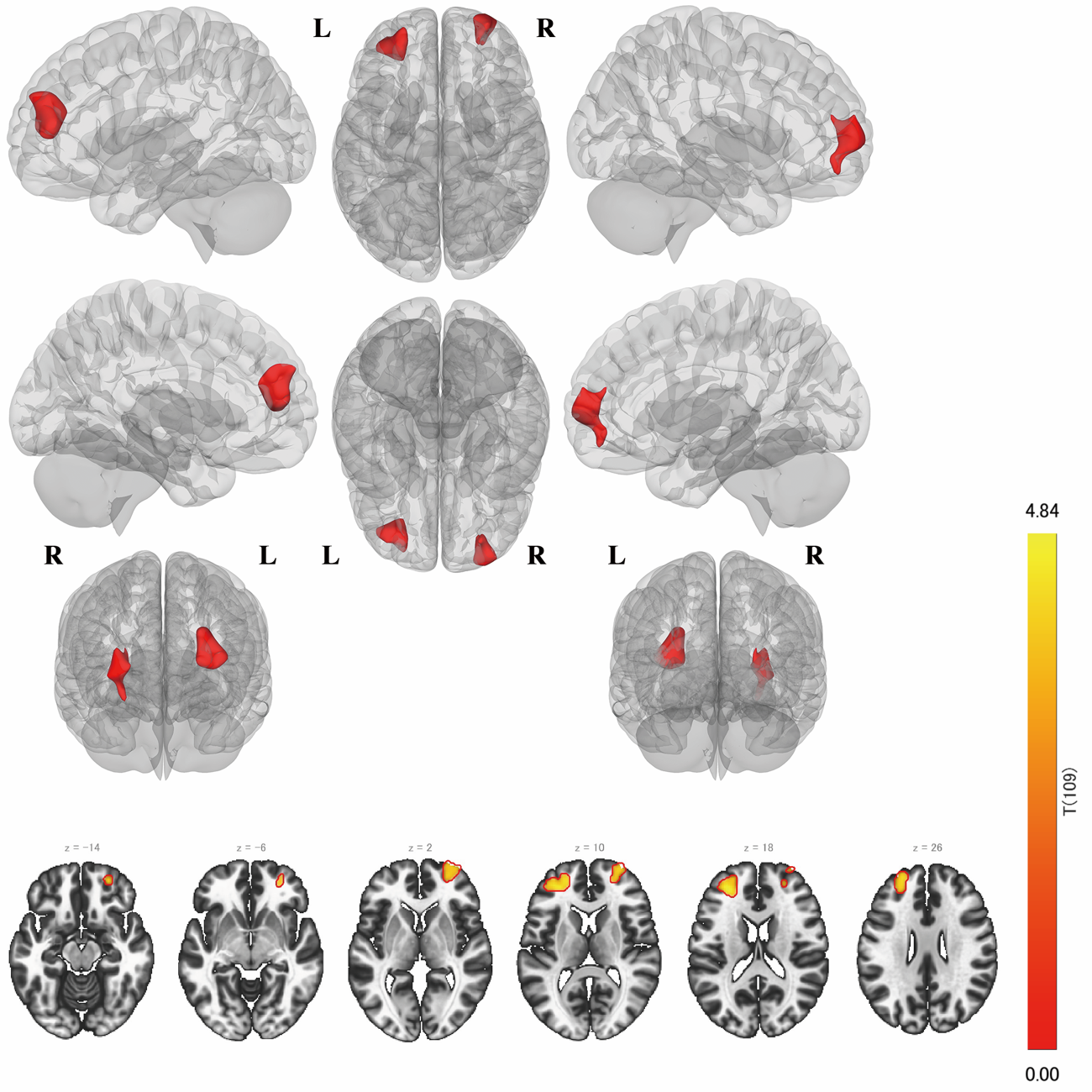


**Supplementary Figure 2. Result of the seed-to-voxel analysis on the cerebellum right crus 1.**

It yielded bilateral dorsolateral clusters. According to these to supplementary figures, the functional connectivity is increased between cerebellum and lateral prefrontal cortices, not medial.
